# Training medical students in opioid overdose prevention and response: Comparison of In-Person versus online formats

**DOI:** 10.1080/10872981.2021.1994906

**Published:** 2021-11-03

**Authors:** Tabitha E. H. Moses, Jessica L. Moreno, Mark K. Greenwald, Eva Waineo

**Affiliations:** a School of Medicine, Wayne State University, Detroit, MI, USA; b Beaumont Health, Southfield, MI, USA

**Keywords:** Medical education, online learning, opioid overdose, medical training

## Abstract

Medical education has increasingly shifted towards replacing large lectures with a combination of online and smaller in-person group sessions. This study compares the efficacy of a virtual Opioid Overdose Prevention and Response Training (OOPRT) for first-year medical students with an identical in-person training. During their first unit of medical school, students in the class of 2023 (cohort 1) received OOPRT in-person and students in the class of 2024 (cohort 2) received training via Zoom. Aside from the delivery format, trainings were identical. Both cohorts completed identical surveys at medical school entry and post-training to evaluate knowledge and experiences using the Opioid Overdose Knowledge Scale, Opioid Overdose Attitudes Scale, Medical Conditions Regard Scale, and Naloxone Related Risk Compensation Beliefs. Of 430 students, 84.2% (362: 124 in cohort 1; 238 in cohort 2) completed baseline and post-training surveys. Students reported significantly improved opioid overdose knowledge and attitudes in all 4 knowledge and 3 attitudes subscales after training. Only one outcome differed by training type: knowledge of opioid overdose signs. Cohorts did not differ in opinions of training; 97.2% enjoyed it and 99.4% believed future classes should receive it. Medical students’ attitudes and knowledge significantly improved after OOPRT; only one of 13 outcomes showed a cohort difference. There were no differences in enjoyment, indicating that switching to virtual learning does not undermine the learning experience. Further studies are needed to confirm that these results can be extended to other medical school topics where small group interactive discussion is preferred.

## Introduction

Over the past decade there has been a shift in undergraduate medical education (UME) as institutions minimized large lecture classes in favor of small group sessions. This shift has led to increased use of online education to replace larger lectures [1–3]. Growing research into the efficacy of online education, especially in medical settings, suggests that when online classes replace larger lectures it does not negatively impact the student learning experience [2,4,5].

Despite significant work in medical education research, few investigations address how traditionally smaller interactive classes and trainings can translate to a virtual format. Although initial studies are positive and suggest that online versions of these trainings lead to similar knowledge gains [6], there is a need for more research. Many benefits of these small group sessions are considered to arise from dynamic interactions between students, which do not immediately translate well to a virtual setting [7–9]. Nonetheless, in 2020 many institutions were forced to shift to virtual formats [10–12]. Given the interactive nature often necessitated by small group sessions, the efficacy of online formats is uncertain [13].

Prior research evaluating the shift from in person to online lectures has shown that the topic of the course does not tend to alter the effects of the different instructional methods [2,4,5]. As a result of these prior findings, we believe that it will be possible to broadly extrapolate the outcomes associated with virtual training in one topic across multiple fields. In this study we compare the efficacy of a synchronous online versus an in-person Opioid Overdose Prevention and Response Training (OOPRT). Despite the opioid overdose focus, we believe these findings will translate to other topics in UME.

### Aims and hypothesis

The present study examines the efficacy of OOPRT conducted via a synchronous online platform (Zoom) versus in-person. The same training was provided by the same facilitator to two different first-year classes of medical students. Our first aim is to establish whether there are differences in the impact of OOPRT on knowledge and attitudes towards opioid overdose depending on training format. We hypothesize that both trainings will improve knowledge and attitudes. Our second aim is to establish whether there is a difference in how the two training formats affected students’ attitudes toward harm reduction and naloxone distribution. We hypothesized that both forms of training would improve students’ understanding of these activities. Our third aim was to explore whether the training format had an impact on students’ enjoyment of and belief in the utility of training. We hypothesize that students who received OOPRT via the synchronous online format would be less engaged and therefore find training less enjoyable than students who received the training during the in-person group session.

## Methods

### Participant selection

This analysis was conducted using data from two larger, ongoing projects examining longitudinal effects of varying curricula related to substance use disorders (SUDs). The studies were designed to track the Class of 2023 and Class of 2024 respectively, throughout UME to monitor changes in attitudes and behaviors surrounding substance use, SUD treatment, overdose response, and harm reduction. Both studies received IRB review and exemption and all students were provided with an information sheet detailing the purpose of the study and the ways in which they could opt out of participation.

Cohort 1 consists of students in the Class of 2023. At matriculation, all medical students (N = 296) were asked to complete a baseline survey between August 15 and 23 September 2019. Approximately 1-month later, 50% of the class was randomly selected to receive in-person OOPRT (the other 50% received training in year 3). Cohort 1 received training during September 2019 in a classroom setting in 4 groups of 30–40 students.

Cohort 2 consists of students in the Class of 2024. At matriculation, all medical students (N = 298) were asked to complete a baseline survey between July 16 and 2 August 2020. All students received OOPRT in their first year via Zoom, in two groups (~150 per group) in September 2020. Immediately following training both cohorts received a post-training survey.

### Training

The OOPR training was developed by an experienced pharmacist specializing in SUDs and harm reduction education, who also served as the faciliator for the training. Details of the curriculum development can be found in Moses *et al*., 2021 [14]. The training is designed to be flexible and updatable based on new evidence in the field; however, there were 9 consistent overarching competency-based goals focusing on knowledge of: opioid overdose identification, opioid overdose risk factors, opioid overdose response, naloxone use, harm reduction, naloxone access laws, gGood Samaritan laws, myth busting, and stigma reduction. The in person and virtual trainings were designed to be as similar as possible, with these competency-based training goals in mind. All trainings, regardless of format, lasted for 1-hour.

For the in person format, the training occured in groups of ~30-40 students. Students were given a one-page handout outlining opioid overdose signs, checking for patient responsiveness, recommendations for making a 911 call, and how to place a person in the recovery position, perform rescue breathing, and administer naloxone. The facilitator led students in discussion through probing questions about opioid overdose pathophysiology, risk factors, signs, and response. Students were then divided into small groups of 4–6 to practice with Nasal Narcan™ demonstration devices. During the final portion of the training, the facilitator asked students what they had heard about naloxone use, harm reduction, and SUDs and focused on ‘myth-busting’ common concerns appearing in lay literature and media. The lecture closed by reviewing Good Samaritan laws around OOPR in the State of Michigan and discussing concerns some people may have when considering whether to call 911 during an overdose.

The goal was to ensure the virtual training was as similar as possible to the in-person training, while also working towards the same competency-based goals. The structure of the training was the same in that the facilitator used probing question and answer style questions to lead discussion on these topics; students were able to respond via the Zoom chat function and the facilitator read and explained each distinct response. There were 3 key differences in the online format: group size, practice naloxone administration, and presentation. Students receiving the virtual training did so in groups of ~150 per training, it was decided that this larger group would not negatively impact engagement because the virtual format only showed the facilitator’s image and did not allow students to see how many others were in the training. Logisitical barriers meant that it was not possible for students in the virtual training to practice naloxone administration with the Narcan™ demonstration devices. Finally, due to the format of the virtual setting, the facilitator showed a PowerPoint presentation as she spoke to outline key points of the training. This presentation contained no additional content beyond that which the facilitator discussed and was included to provide a visual stimulus for students in the virtual setting.

Although it was not feasible for the trainings to be completely identical, we believe that the content and general delivery was the same in both formats. Importantly, the training goals and outcomes measured were identical across both settings, which allows for comparison between the two training groups.

### Measures

Both cohorts completed surveys at medical school entry and immediately post-training, which included questions regarding previous healthcare experiences, experiences working with people with SUDs, and use of naloxone. To measure knowledge and attitudes we used a series of validated assessments: Opioid Overdose Knowledge Scale (OOKS), Opioid Overdose Attitudes Scale (OOAS) [15], Medical Conditions Regard Scale for SUDs (MCRS) [6], and Naloxone Related Risk Compensation Beliefs (NaRRC-B) [16]. For details on these assessments and their scoring, see Moses *et al*., 2020 [17]. The MCRS can also be calculated as a total score across all 11 statements to identify the general attitudes towards patients with SUDs [18]. The post-training survey included these 4 assessments along with questions about the how the students perceived the effectiveness of the OOPRT, and their general experiences of the training (Supplemental Figure S1 lists the subjective post-training questions). These subjective experience questions were primarily binary yes/no questions but students were given the opportunity to expand upon their answers in an optional open-ended question at the end of the post-training survey.

### Data analysis

All participants who completed the OOPRT and baseline and post-training surveys were included in the analysis. Descriptive data are presented as mean ± one standard deviation unless otherwise specified. The criterion of *p* < .05 was used to reject the null hypothesis (SPSS v.26).

Independent t-tests and chi-square analyses were used to explore group differences in responses. Repeated Measures (RM) ANOVA with cohort (in-person vs. online training) as the between-subjects variable was used to identify changes in response to training and the impact of training type on responses to the OOKS, OOAS, MCRS, and NaRRC-B, as well as any differences in the binary variables measuring subjective enjoyment or perception of utility of the training itself.

## Results

### Participant characteristics

Of the 430 students across both cohorts who received training, 362 (84.2%) completed both the baseline and post-training surveys. 124 students (34.3%) were from Cohort 1 and 238 (65.7%) were from Cohort 2. Mean (SD) age was 23.3 (2.4) years and 50.3% (182) were male. Over half (64.4%) had worked in a healthcare setting prior to medical school 57.0% believed they may have seen a patient with OUD when volunteering or working in healthcare before medical school. Table 1 presents demographic data by cohort; there were no significant demographic differences between cohorts.

**Table 1.** Demographic characteristics at baseline and responses to training for each cohort (Cohort 1: Class of 2023, in-person training; Cohort 2: Class of 2024, virtual training). Comparisons were made for these variables between each cohort to identify any baseline or post-training differences using either t-tests (for continuous variables) or chi-square tests (for dichotomous variables). No significant differences were found (*p* < .05) between the two cohorts for any of the reported baseline on training response variables

		Cohort 1 (N = 124)	Cohort 2 (N = 238)	Test statistic (t-test or Chi-square)	p-value
**Demographics**	Age	23.4 ± 2.2	23.3 ± 2.5	0.025	0.980
	Gender (female)	62 (50.0%)	118 (49.6%)	0.006	0.940
	Race (white)	77 (62.1%)	127 (53.4%)	2.817	0.093
**Clinical Characteristics**	Worked in healthcare prior to medical school	76 (61.3%)	157 (66.0%)	0.777	0.378
	Seen a patient with OUD	45 (59.2%)	134 (56.3%)	1.388	0.708
	Attended a naloxone training	11 (8.9%)	22 (9.2%)	0.014	0.907
**Experience with Substance Use Disorders (SUDs)**	Know someone with a SUD	60 (48.4%)	102 (42.9%)	1.008	0.315
	Seen someone overdose	15 (12.1%)	25 (10.5%)	0.210	0.646
	Know someone who overdosed	33 (26.6%)	54 (22.7%)	0.688	0.407
**Training Interest and Enjoyment**	Interested in receiving OOPRT	117 (94.4%)	233 (97.9%)	3.195	0.074
	Enjoyed the OOPRT	118 (95.2%)	234 (98.3%)	3.027	0.082
	Future classes should receive OOPRT	122 (98.4%)	238 (100.0%)	3.860	0.117
	Students should receive a naloxone kit	122 (98.4%)	235 (98.7%)	0.074	0.785

We also examined the knowledge and attitudes students had towards opioid overdose prior to entering medical school. At baseline, there were significant differences between cohorts in opioid overdose knowledge and in attitudes towards patients with SUDs. Cohort 1 demonstrated more knowledge of opioid overdose risk factors (7.9 ± 1.6 vs. 7.1 ± 2.1; t = 3.758, *p* < 0.001) and naloxone use (9.6 ± 2.7 vs. 8.9 ± 2.3; t = 2.561, *p* = 0.011) and felt more competent to respond to an opioid overdose (2.6 ± 0.66 vs. 2.4 ± 0.74; t = 2.749, *p* = 0.006) than Cohort 2. Conversely, Cohort 2 demonstrated slightly better attitudes towards patients with SUDs, as measured by total MCRS score (49.1 ± 6.3vs. 46.8 ± 6.8; t = 3.244, *p* = 0.001), than students in Cohort 1. There were no other differences between cohorts in their knowledge, views or understanding of harm reduction.

### Training effects on opioid overdose knowledge and attitudes

**Figure 1.**
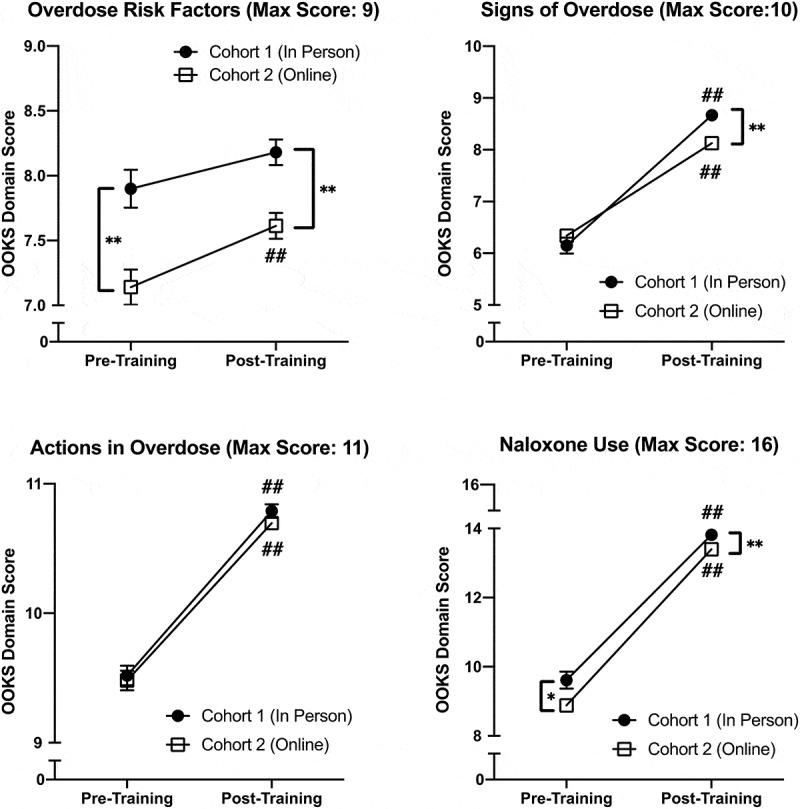
Change in Opioid Overdose Knowledge Scale (OOKS) scores for all 4 domains that showed a significant effect of training across Cohort 1 (n = 124) and Cohort 2 (n = 238). Cohort had a significant effect on response to training in 1 of the 4 domains: signs of an overdose

RM ANOVA with cohort as the between-subjects variable was used to identify effects of training on the 4 OOKS and 3 OOAS domains. There was a significant main effect of time (independent of cohort) on all 4 OOKS domains (Table 2). Figure 1 shows changes over time by cohort for all OOKS domains. One domain showed a significant interaction between cohort and time. For signs of an opioid overdose (*F*(1,360) = 12.83; *p* < 0.001, partial η^2^ = 0.034), Cohort 1 demonstrated greater mean knowledge improvement (6.15 ± 1.71 to 8.67 ± 0.95) than Cohort 2 (6.34 ± 1.67 to 8.13 ± 1.48).

**Table 2.** Results of RM ANOVA across the 2 timepoints (baseline, post-training) with cohort as the between subjects factor for each domain of Opioid Overdose Knowledge Scale (OOKS), Opioid Overdose Attitudes Scale (OOAS), and Naloxone Related Risk Compensation Beliefs (NaRRC-B)

		F (1,360)	p	Partial η^2^
**OOKS Opioid Overdose Knowledge Domains**	Overdose risk factors	**11.65**	**<0.001**	**0.031**
	Signs of overdose	**431.29**	**<0.001**	**0.545**
	Actions to take in overdose	**374.93**	**<0.001**	**0.510**
	Naloxone use	**850.08**	**<0.001**	**0.702**
**OOAS Opioid Overdose Attitude Domains**	Competencies	**1590.07**	**<0.001**	**0.815**
	Concerns	**246.37**	**<0.001**	**0.406**
	Readiness to intervene	**15.50**	**<0.001**	**0.041**
**NaRRC-B** **Naloxone Related Risk Compensation Beliefs**	Opioid/heroin users will use more opioids/heroin if they know they have access to naloxone	**123.48**	**<0.001**	**0.255**
	Opioid/heroin users will be less likely to seek out treatment if they have access to naloxone	**99.99**	**<0.001**	**0.217**
	Providing naloxone to overdose victims sends the message that I am condoning opioid misuse	**39.57**	**<0.001**	**0.099**
	There should be a limit on the number of times one person receives naloxone to reverse an overdose	**44.70**	**<0.001**	**0.110**
	Naloxone is enabling for drug users	**55.46**	**<0.001**	**0.133**

* indicates significant difference between the two groups at that time point; *p < 0.05, **p < 0.01

# indicates significant change from baseline for that group; #p < 0.05, ##p < 0.01

**Figure 2.**
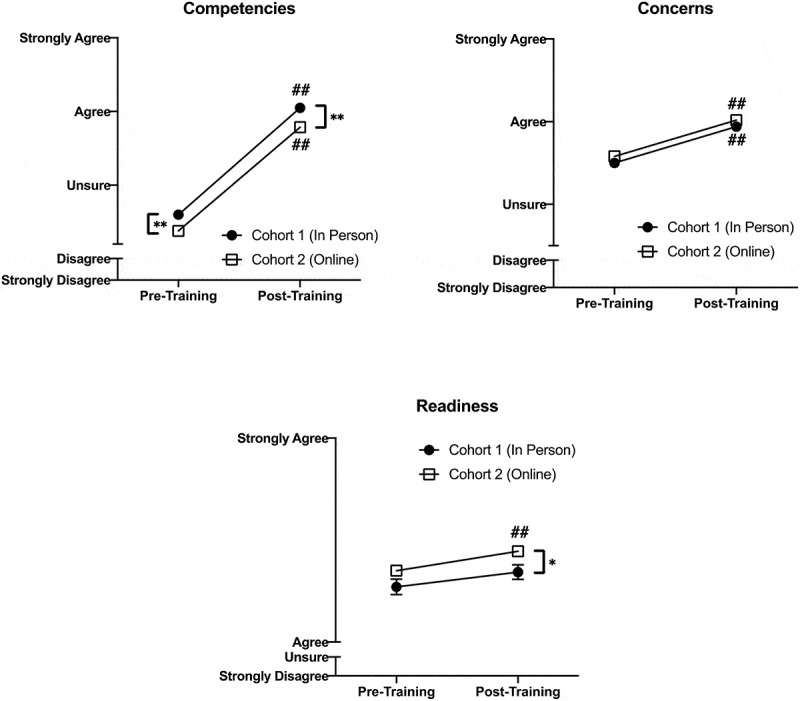
Change in Opioid Overdose Attitudes Scale (OOAS) scores for all 3 domains that showed a significant effect of training across Cohort 1 (n = 124) and Cohort 2 (n = 238). Cohort had no significant effect on response to training in any domain

There was also a significant main effect of time (independent of cohort) for all 3 OOAS domains; scores improved after training (Table 2). Figure 2 shows changes over time by cohort for all OOAS domains. There was no interaction between cohort and time for any OOAS domain.

* indicates significant difference between the two groups at that time point; *p < 0.05, **p < 0.01

# indicates significant change from baseline for that group; #p < 0.05, ##p < 0.01

### Training effects on attitudes towards harm reduction

To explore effect of training type on attitudes towards harm reduction and patients with SUDs we used RM ANOVA with cohort as the between-subjects variable. We found a significant main effect of time (independent of cohort) on attitudes toward patients with SUD measured by total MCRS score; student attitudes generally improved after training (*F*(1,360) = 22.55; *p* < 0.001, partial η^2^ = 0.059). There was no interaction between cohort and time.

**Figure 3.**
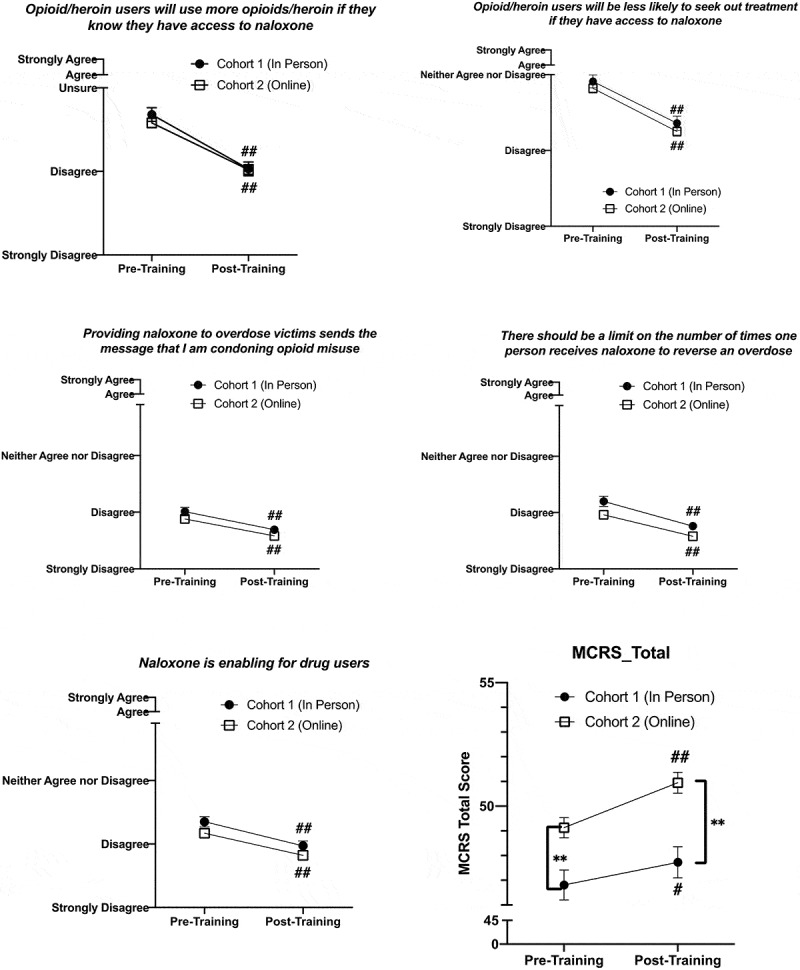
Change in all 5 Naloxone Related Risk Compensation Beliefs (NaRRC-B) statement scores and total Medical Conditions Regard Scale score, which all showed a significant effect of training across Cohort 1 (n = 124) and Cohort 2 (n = 238). Cohort had no significant effect on response to training in any domain

We also found a significant effect of training on attitudes toward naloxone use and distribution. Training improved responses to all 5 NaRRC-B statements with students being more likely to disagree with all statements after training (Table 2). There was no interaction between cohort and time on attitudes towards naloxone. Figure 3 shows changes over time by cohort for the MCRS total score and 5 NaRRC-B statements.

* indicates significant difference between the two groups at that time point; *p < 0.05, **p < 0.01

# indicates significant change from baseline for that group; #p < 0.05, ##p < 0.01

### Student attitudes towards training

Prior to training, students were asked about their interest in receiving OOPRT and 350 (96.7%) indicated they wanted to receive the training. After training, students were asked whether they enjoyed it and 352 (97.2%) stated they did. Almost all (360; 99.4%) believed that future medical school classes should receive a similar training. Table 1 shows these responses by cohort; there was no difference between cohorts.

In the post-training survey, students were provided with an open-ended question for any additional training feedback. A total of 48 students (14 from Cohort 1; 34 from Cohort 2) chose to respond. The majority of these responses were positive. In Cohort 1 there were 2 negative comments, both critiquing the fact that training was required during an exam week. In Cohort 2, there were 2 negative comments about the training: one did not like some specific advice given by the trainer regarding what to say when calling for medical assistance and the other felt that students who had previously worked in healthcare should be exempt from attending this training. Nine comments were specific to the virtual format; 7 of these students lauded the training but hoped there could be an additional in-person session when appropriate for students to have ‘hands-on’ training with naloxone devices. Most notable were comments that demonstrated the students’ engagement with the virtual training such as, ‘*This training was one of the more interesting/engaging Zoom webinars that M1s have had so far*’ and ‘*I thought it was very informative and well done, even in the conference call format of Zoom. Great job to the presenter!*’. These comments demonstrate that students recognize the difficulties of the virtual learning environment but that they felt those problems were mostly overcome.

## Discussion

The goal of this study was to explore the impact of training format (in-person vs. online) on learner outcomes for OOPRT provided to first-year medical students. Results of this study provide strong support for the use of online platforms in medical training; however, they demonstrate that for many students a ‘hands-on’ component is still desired. We believe the specific training content does not constrain the wider implications of the findings. Previous research into teaching methodology and techniques suggests that successful teaching styles and approaches are relatively consistent across disciplines [19–23]. Although the impetus for this comparison stemmed from the rapid shift to online learning associated with the COVID-19 pandemic, the results are far-reaching. Not only is online learning more flexible for students and educators, but the ability to provide these trainings virtually allows for implementation at institutions that may not otherwise have resources to offer them. This is especially important for a training such as OOPRT, which requires an experienced facilitator to be most effective. There is increasing evidence demonstrating that this type of training in harm reduction is necessary in medical education and desired by trainees [24–27]; however, not all institutions have the capacity to introduce this training in-person. This initial evidence demonstrating the comparative efficacy of virtual OOPRT opens the door for more institutions to be able to provide these trainings to their students and staff with minimal additional overhead.

Students in this study were from two consecutive medical school classes. Both groups completed OOPRT as early as possible during their first year. One major strength of this study is the sample size available for comparison. A total of 362 students were included for analysis; student demographics did not differ between cohorts and were aligned with those of first-year medical students across the USA [28]. We did identify some initial differences between the two groups regarding knowledge and attitudes towards opioid overdose and patient with SUDs, which was unexpected as neither group received any exposure to medical school curricular content on SUDs prior to this training. We considered the impact of general changes in pre-medical education, content-relevant media coverage, and other factors that could have impacted this finding, but the exact cause for these differences remains unclear.

The first aim of this study was to evaluate whether training method played a role in student knowledge attainment. Both cohorts showed significant improvement in all domains of opioid overdose knowledge and attitudes toward responding to an opioid overdose. This aligns with findings from studies of similar trainings with medical students, the majority of which found significant improvements in these outcomes [6,24,29]. Additional analyses suggest a potential effect of cohort on one domain of overdose knowledge: signs of an opioid overdose, with Cohort 1 improving more after training than Cohort 2. Although this difference was slightly less than 1 point, the domain has a total possible score of 10, which means that this 1 point translates to an almost 10% difference. This finding was unexpected and inconsistent with the results of another study that compared online versus in-person OOPRT in medical students [6]; however, that study used a modified version of the OOKS that resulted in only 1 unique knowledge score so it is possible that details of the knowledge domains measured by the OOKS were not fully identified. It is unclear why we found this difference. One hypothesis is that during online training students were shown a PowerPoint that included a list of signs of opioid overdose on one slide. During the in-person training the content was identical; however, there was no PowerPoint presentation used alongside the training. Although the trainer still went through the signs of overdose it is possible that including the visual list distracted students from absorbing details of this portion of the discussion.

The second aim of this study was to identify whether training impacted student attitudes towards harm reduction and patients with SUDs, and whether training method impacted these responses. Although initial analyses suggested that training has minimal impact on attitudes towards people with SUDs when evaluating the 11 individual MCRS statements separately [30], here by analyzing attitudes as one combined MCRS total score variable, we do see a small effect. Training resulted in increased total MCRS scores, which translates to general improvement in attitudes towards patients with SUDs. This finding contrasts with some similar studies, which have not found that this type of training impacts these attitudes in medical students [29]; however, other studies of medical residents have found positive effects of training on attitudes towards patients [26,31]. Additionally, when looking at attitudes toward naloxone distribution, training improved attitudes in all 5 areas measured. We found no effect of training method on these outcomes. Regardless of the platform for training, a 1-hr session will not solve the problems of stigma towards patients with SUDs that exist within healthcare; nonetheless, education about the facts and dispelling myths are important first steps in larger anti-stigma initiatives within healthcare facilities. As such, the ability of virtual training to manifest the same level of attitude improvement as in-person training creates opportunities for implementing these trainings in facilities that may lack staff to conduct the trainings themselves.

Our final aim explored how student enjoyment of and engagement in the training differed based on training method. Anecdotal reports suggest that students may feel more engaged in small group classes than large online sessions, and student engagement may play a role in the efficacy of the educational initiative [32–35]. The results of this study are promising. We found no difference in training enjoyment between the two cohorts and most students believed that future medical school classes should receive the training regardless of the format. Notably, several students in Cohort 2 took time to comment about the important role that the facilitator played in keeping their engagement in the virtual session. This demonstrates the importance of attempting to translate the interactive nature of these small group sessions to a virtual platform; an easy way to do this is by using the chat function to engage students. Finally, despite these very positive responses, some students indicated a desire for a ‘hands-on’ portion of the training where possible. This is more specific to the OOPR training, as the in-person trainings included practice with naloxone devices; in a virtual setting this is not possible without distribution of naloxone kits or training devices to all participants prior to the session, and students recognized this lack of skill building. Notably, lack of hands-on training did not impact student confidence or self-perceived readiness to intervene in an opioid overdose so while students may desire this aspect of training it may not be as necessary to learning as previously thought. We must also consider the context of holding a virtual training when in-person sessions were not possible due to COVID-19, and the possibility that more students may prefer in-person sessions when these are able to be offered.

This study is not without limitations. First, data were gathered at one medical school, however, the response rate was high and the class was large and diverse with demographics matching those of first-year medical students nationally [28]. Second, all data were self-reported, which may result in a bias toward social desirability, although we attempted to mitigate this concern by assuring students that responses were confidential. Third, the overarching research projects were not designed for this comparison, resulting in unequal groups; we believe the generally large sample size and minimal group differences reduce the impact of this limitation. Fourth, the training occurred at one school with the same facilitator and may not be generalizable to all settings; nonetheless, we believe that our consistent findings with this training across multiple class years and the fact that our outcomes align with those found after similar trainings elsewhere reduces this concern [6,14,29,30]. Fifth, the training was focused on one specific topic and may not be generalizable to other topics; however, prior education research demonstrates that successful teaching techniques are typically effective across multiple domains, suggesting that the single topic nature of this research does not limit the generalizability of the results [19,23,36].

Our results suggest that implementing a synchronous online OOPR training results in similar educational outcomes to conducting the same training in an in-person, small group setting. Furthermore, the larger class size and online format did not decrease student engagement with or enjoyment of the training itself. Findings from this initial study provide empirical support for the transition to online education in medicine and provide some relief for those concerned that the necessary shift to virtual formats for small group sessions as a result of COVID-19 may have negatively impacted medical education. Encouragingly, almost all students across both groups enjoyed training and wanted to improve their knowledge on this topic. Although these results are important for medical schools such as our own, they may be especially vital for smaller, more geographically remote institutions that do not have the faculty to teach certain trainings. These findings suggest that incorporating a virtual training from a remote facilitator may be just as beneficial to student learning. Next steps include identifying the long-term effects of OOPR training on these outcomes and whether the method of training delivery impacts long-term outcomes. Finally, it will be important to analyze how these trainings and the associated improvements translate to clinical and volunteering behavior among students in both cohorts.

## Supplementary Material

Supplemental Material
