# Supplementary material for: Training medical students in opioid overdose prevention and response: Comparison of In-Person versus online formats
Source: Med Educ Online. 2021 Nov 3;26(1):1994906. doi: 10.1080/10872981.2021.1994906 (PMC8567883; doi:10.1080/10872981.2021.1994906)
Supplement: Supplemental Material [file ZMEO_A_1994906_SM9097.docx]

**Supplemental Figure 1:** Questions asked to students in the post-training survey regarding their enjoyment of and experiences with the Opioid Prevention and Response Training (OOPRT)

Q7.1 Did you enjoy the opioid overdose prevention training?

- Yes (1)
- No (2)

Q7.2 Do you think future classes should receive opioid overdose prevention training?

- Yes (1)
- No (2)

Q7.3 Did you take the pre-training survey (sent by Dr Waineo)?

- Yes (1)
- No (2)

Q7.4 Did the pre-training survey help in your understanding of opioid overdose prevention?

- Yes (1)
- No (2)

Q7.5 Did this post-training survey help in your understanding of opioid overdose prevention?

- Yes (1)
- No (2)

Q7.6 Do you think all students attending the training should receive a naloxone kit?

- Yes (1)
- Maybe (3)
- No (2)

Q7.7 If the school provided kits to students, should there be a cost to the students?

- Yes, students should pay for the full cost of the kits (1)
- Yes, students should pay for a subsidized kit (2)
- No (3)

Q7.8 Please add any comments you have about the opioid overdose prevention training.
